# Effects of cell phenotype and seeding density on the chondrogenic capacity of human osteoarthritic chondrocytes in type I collagen scaffolds

**DOI:** 10.1186/s13018-020-01617-6

**Published:** 2020-03-30

**Authors:** Chenxi Cao, Yujun Zhang, Yanqi Ye, Tiezheng Sun

**Affiliations:** 1 Arthritis Clinic and Research Center, Peking University People’s Hospital, Beijing, 100044 People’s Republic of China; 2 Institute of Sports Medicine, Peking University Third Hospital, Beijing, 100044 People’s Republic of China; 3 The Institute of Clinical Molecular Biology and the Central Lab, Peking University People’s Hospital, Beijing, 100044 People’s Republic of China

**Keywords:** Osteoarthritis, MACI, Cellular phenotype, Seeding density, Type I collagen scaffolds

## Abstract

**Objective:**

Matrix-associated autologous chondrocyte implantation (MACI) achieves good clinical efficacy in young patients with focal cartilage injury; however, phenotypic de-differentiation of chondrocytes cultured in monolayer and the treatment of older OA patients are still challenges in the field of cartilage tissue engineering. This study aimed to assess the in vitro re-differentiation potential and in vivo chondrogenic capacity of human OA chondrocytes inoculated into collagen I scaffolds with different cellular phenotypes and seeding densities.

**Methods:**

OA chondrocytes and articular chondrocyte-laden scaffolds were cultured over 2 weeks in in vitro. Reverse transcriptase quantitative polymerase chain reaction (RT-qPCR) and histological staining were used to detect the mRNA expression profiles and extracellular matrix secretion of chondrocyte-specific markers. OA chondrocyte-laden collagen I scaffolds with different cellular phenotypes, and seeding densities were implanted into SCID mice over 4 weeks to evaluate the chondrogenic capacity in vivo.

**Results:**

Increased COL2a1, ACAN, COMP, SOX9, and BMP2 expression levels and decreased COL1a1, VCAN, MMP13, and ADAMTS5 amounts were observed in OA chondrocytes seeded in collagen I scaffolds; Implantation of phenotypically superior OA chondrocytes in collagen I scaffolds at high density could improve the chondrogenic capacity of human OA chondrocytes, as confirmed by RT-qPCR assessed gene expression patterns in vitro and histological evaluation in vivo.

**Conclusions:**

Freshly isolated chondrocytes from OA patients could be a source of replacement for articular chondrocytes being commonly used in MACI. Implantation of phenotypically superior OA chondrocytes in collagen I scaffolds at high density could be a promising tool for the treatment of elderly OA patients.

## Introduction

Full-thickness articular cartilage defects have little potential for self-repair based on poor vascularity and limited spontaneous healing ability [1]. Untreated cartilage defects are associated with pain and swelling, leading to immobility and resulting in osteoarthritis (OA) [2]. In the past two decades, several reparative procedures for the cartilage have been developed, and early surgical intervention for symptomatic cartilage lesions currently attracts increasing attention. Matrix-associated autologous chondrocyte implantation (MACI) is one of the most important methods, especially for critically sized defects [3]. MACI is based on the isolation of chondrocytes from minor load-bearing areas of the knee and monolayer culture in vitro to reach an adequate density before cell implantation on porous scaffolds into the cartilage defect [4]. Previous findings suggest that the MACI technology achieves good clinical efficacy in young patients with focal cartilage injury [5]. Ulrich Schneider et al. performed type I collagen hydrogel-based autologous chondrocyte implantation (CaReS) in 116 young patients with knee joint cartilage injury, and their experiment shows matrix-associated ACI employing the CaReS technology for the treatment of chondral or osteochondral defects of the knee is a safe and clinically effective treatment that yields significant functional improvement and improvement in pain level [6]. However, phenotypic de-differentiation of chondrocytes cultured in monolayer and the treatment of older OA patients are still challenges in the field of cartilage tissue engineering [7].

The present feasible method for maintaining phenotypic stability of cultured chondrocytes is cell transfer into 3D environment, and the de-differentiation process may be reversed [8]. In vitro culture of chondrocytes under 3D conditions may favor phenotypic retention; however, it is less efficient for cell proliferation in yielding enough amounts compared with monolayer culture [9]. Monolayer culture amplification could effectively increase the cell seeding density, which also serves as an important parameter determining the outcome of MACI [10]. Therefore, improved cell phenotype and density for chondrocytes during monolayer amplification constitute a pair of conflicting variables that both affect the ultimate outcome of MACI. How they affect the final outcome and which of them is more important in cartilage tissue engineering remain largely unknown. Whether MACI could be used to treat cartilage damage in elderly OA patients is unclear, as well as the chondrogenic capacity of autologous OA chondrocytes.

This study aimed to assess the in vitro re-differentiation potential and in vivo chondrogenic capacity of human OA chondrocytes inoculated into type I collagen scaffolds with different cellular phenotypes and seeding densities. Ultimately, the current study may contribute to a better control of phenotypic stability in MACI and help optimize cartilage tissue engineering for the treatment of elderly OA patient.

## Materials and methods

### Osteoarthritic chondrocyte isolation and passage

Cartilage samples were collected from 26 patients (5 males and 21 females; 65.4 ± 4.9 years, ranging from 56 to 79 years) in Peking University People’s Hospital with primary OA undergoing total knee arthroplasty. All cartilage samples were taken from the lower level mechanical loading and non-damaged areas of the joint and mechanically sliced into 2–5 mm^3^ pieces, especially the lateral tibial plateau and the lateral femoral condyle, because the lateral compartment of the OA patient with varus deformity is often not seriously damaged. All patients had signed informed consent before surgery, and all procedures were approved by the Ethics Committee of the Peking University People’s Hospital. Then, the slices were enzymatically digested for 6–10 h with 0.2% type II collagenase (Gibco, USA). Chondrocytes were collected and resuspended in 10% FBS-DMEM/F12 medium (Corning, USA) containing 1 g/L penicillin-streptomycin (Invitrogen, USA) at 37 °C in a humid environment with 5% CO_2_. Passage 1 (P1) to (P5) chondrocytes were selected from culture dishes for subsequent experiments. All patients signed informed consent before surgery, and all procedures were approved by the Ethics Committee of Peking University People’s Hospital.

### Glycosaminoglycan Elution assay

Glycosaminoglycan (GAG) was extracted from 5 × 10^5^ monolayer-cultured OA chondrocytes at each passage (P1, P3, and P5) and assessed by the modified 1,9-dimethyl-methylene blue (DMMB)-based spectrometric method using a GAG kit (ToYong Bio, China) according to the manufacturer’s instructions. The contents of eluted proteoglycan were determined by measuring absorbance at 656 nm on a spectrophotometer. Each test was performed in triplicate.

### Histochemical and immunofluorescent staining of monolayer-cultured OA chondrocytes

For histochemistry, monolayer-cultured OA chondrocytes at each passage were fixed with 4% paraformaldehyde for 20 min in the cell culture dish, then they were stained with safranin-O (0.1%; Sigma, USA) for 20 min and toluidine blue (1%; Sigma, USA) for 1 h. For immunofluorescence, the cell samples were fixed with 4% paraformaldehyde for 20 min in the cell culture dish and incubated in 300 μl 0.2–0.3% Triton-X100 for 30 min to break the cell membrane, then added goat serum blocking solution to each well for 1 h. Finally, the cells were incubated with rabbit anti-collagen I or II antibodies (1:100 dilution; Abcam, USA) respectively, overnight at 4 °C and further incubated with horseradish peroxidase-conjugated secondary antibodies (ZSGB-BIO, China) for 1 h at room temperature in the dark. At last, the samples were counterstained with DAPI (1:1000 dilution; Sigma, USA) for 10 min, and images were captured under a fluorescence microscope (LEICA, USA).

### Scaffold preparation and 3D culture

For type I collagen scaffolds, 0.5 ml of 6.32 mg/ml collagen type I solution (Arthro-Anda Tianjin Biologic Technology Company, China) was mixed with 0.4 ml 2× neutral buffer solution and 0.1 ml FBS. The specific preparation process is as follows: first, chondrocytes (P1, P3, P5) were resuspended in a 2 × neutral buffer solution, adjusted the concentration to a low concentration of 5 × 10^5^ cells/0.4 ml or a high concentration of 2 × 10^6^ cells/0.4 ml, add 0.4 ml of the cell suspension into a 24-well plate, and then add 0.5 ml of type I collagen solution and 0.1 ml of FBS to each well using a sterile liquid stirring bar to slowly stir to fully mix the three solutions and cells. Finally, the cells were seeded into scaffolds at the density of 5 × 10^5^ cells/ml or 2 × 10^6^ cells/ml. After initial gelatinization, the generated collagen type I scaffolds were allowed to polymerize for 10–15 min at 37 °C.

### Quantitative real-time PCR

Total RNA was obtained from monolayer chondrocytes (P1, P3, and P5) or 3D scaffolds after 2 weeks of culture with the total RNA kit (Omega, USA). For extracting cellular RNA from a type I collagen scaffold, the 1 ml scaffold was cut into small pieces, and 2 ml of 1 U/ml Collagenase NB 4 Standard Grade (Serva, Germany) solution was added and digested at 37 °C for 1 h. The digestion solution was then transferred to a centrifuge tube, centrifuged at 1000 rpm for 10 min, and cell lysate was added to extract total RNA. cDNA was synthesized from 1 μg total RNA with a qPCR-RT kit (Toyobo, Japan). Next, qRT-PCR was performed on a MiniOpticon qPCR (Bio-Rad, USA) with SYBR Green PCR Master Mix (Toyobo, Japan). GAPDH expression was used to normalize individual mRNA expression levels. The primers used are listed in Supplementary Table 1. Data were analyzed by the comparative cycle threshold method (ΔΔCt), and results were expressed as 2^-ΔΔCt^. All tests were performed in triplicate.

**Table 1:** Primer sequences used in real-time quantitative PCR

Gene	Primer sequences	Length(bp)
COL2-a1	**F**:5′GGACTTTTCTCCCCTCTCT	104
	**R**:5′GACCCGAAGGTCTTACAGGA	
COL1-a1	**F**:5′AGAGGAAGGAAAGCGAGGAG	120
	**R**:5′GGACCAGCAACACCATCTG	
ACAN	**F**:5′AGAATCAAGTGGAGCCGTGT	116
	**R**:5′GGGTAGTTGGGCAGTGAGAC	
VCAN	**F**:5′TGTTCCTCCCACTACCCTTG	122
	**R**:5′CTTCCACAGTGGGTGGTCTT	
COMP	**F**:5′TCAGGACTCTCGGGACAACT	117
	**R**:5′CTGTCAGGGACTCCGTCATT	
BMP-2	**F**:5′GTCCTGAGCGAGTTCGAGTT	115
	**R**:5′AGTGCCTGCGATACAGGTCT	
SOX-9	**F**:5′GGAATGTTTCAGCAGCCAAT	115
	**R**:5′TGGTGTTCTGAGAGGCACAG	
MMP-13	**F**:5′GCAGTCTTTCTTCGGCTTAGAG	100
	**R**:5′GTATTCACCCACATCAGGAACC	
ADAMTS-5	**F**:5′TGTGAAGAGACCTTTGGTTCC	111
	**R**:5′TTCTGTGATGGTGGCTGAAG	
GAPDH	**F**:5′GTCTCCTCTGACTTCAACAGCG	131
	**R**:5′ACCACCCTGTTGCTGTAGCCAA	

### Cell viability assay

LIVE/DEAD® Viability Kit (Thermo Fisher Scientific, USA) was used to assess the viability of OA chondrocytes(P3) in scaffolds in vitro to verify whether the scaffold material is cytotoxic. The working solution was prepared according to the manufacturer’s instructions. Then, 200 μl working solution was added to each sample and incubated at 37 °C in a humid environment with 5% CO_2_ for 10 min. Images were acquired under a fluorescent microscope.

### Establishment of an animal model of ectopic chondrogenesis

A total of 9 male CB17-SCID mice (age, 6–8 weeks) were provided by Beijing Vital-River Laboratory Animal Technology Company and maintained in a specific pathogen-free (SPF) environment. All animal experimental procedures, including animal purchase, feeding, operation, and execution were approved by the Experimental Animal Ethics Committee of Peking University People’s Hospital. After anesthetizing mice with a small animal sevoflurane inhalation anesthesia machine, a 1-cm incision was made on the mouse back. After separating the subcutaneous fascia and fat with surgical forceps, type I collagen scaffolds with different cell phenotypes and seeding densities of chondrocytes were implanted into subcutaneous pockets. Four weeks after implantation of type I collagen scaffold, the mice were euthanized with CO_2_, and the samples were isolated and processed for histological examination. Nine male CB17-SCID mice (age, 6–8 weeks) were randomly divided into 3 groups with 3 for each group. The first group was used to compare the differences between P1, P3, and P5; the second group was used to compare the LP3 and HP3; and the third group was used to compare between LP1 and Hp3. Each mouse in each group was implanted with all the samples of the group in the back. For example, each mouse in the first group was implanted with P1, P3, and P5 samples on the back. Other groups were implanted in a similar manner.

### Histological analysis of scaffolds

After 4 weeks, mice were sacrificed, and samples were harvested. Serial paraffin sections (4 μm) were routinely prepared. H&E staining was performed to evaluate cell morphology. GAGs were visualized by staining with 0.1% safranin-O and 1% toluidine blue. The synthesized collagen types II and I were detected by immunohistochemical staining. Briefly, paraffin sections were routinely dewaxed with xylene solution and gradient alcohol and were incubated with 3% H_2_O_2_ for 10 min to inhibit endogenous peroxidase; next, the sections were heated with a pH 6.0 citrate buffer in 98 °C water bath for 20 min for antigen retrieval. Then, the sections were incubated with 10% goat serum for 1 h at room temperature for non-specific antigen blocking. Next, the sections were incubated with rabbit anti-collagen II or I primary antibodies (1:100), respectively, overnight at 4 °C. Subsequently, sections were incubated for 30 min at room temperature with horseradish peroxidase-conjugated secondary antibodies (ZSGB-BIO, China). Finally, the sections were counterstained with hematoxylin. Data were assessed by 3 blinded observers with the Bern Score system, which evaluates uniformity and intensity of safranin O staining, the distance between cells, the amounts of matrix produced, and cell morphology. Each item could be given a maximum of 3 points, for a maximum of 9 points.

### Statistical analysis

**Fig. 1:**
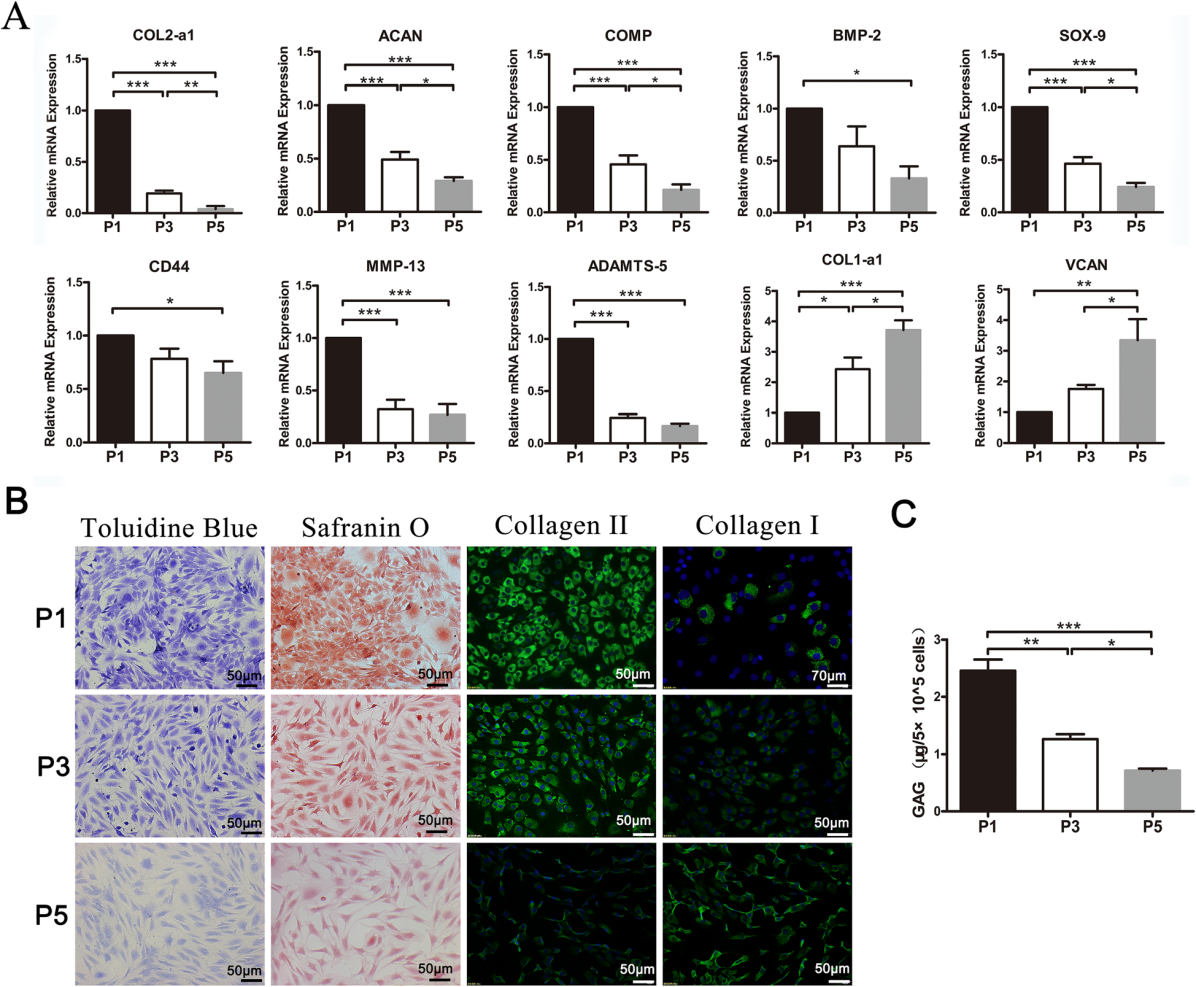
Specific gene expression profiles and extracellular matrix staining of monolayer cultured human OA chondrocytes. **a** Real-time PCR to quantify specific gene expression profiles of monolayer cultured OA chondrocytes among P1, P3, and P5 (*n* = 3 in each group). Significance values were *p < 0.05, **p < 0.01, and ***p < 0.001; error bars indicated standard errors of the means. **b** Extracellular matrix staining of monolayer cultured human OA chondrocytes. Toluidine blue, safranin O, and immunofluorescence staining for type II and I collagen in OA chondrocytes cultured in monolayer. **c** Cell glycosaminoglycan released into the medium. P1–P5 cultures were analyzed by the DMMB assay (*n* = 3 in each group). *p < 0.05, **p < 0.01, and ***p < 0.001; data are mean ± standard

**Fig. 2:**
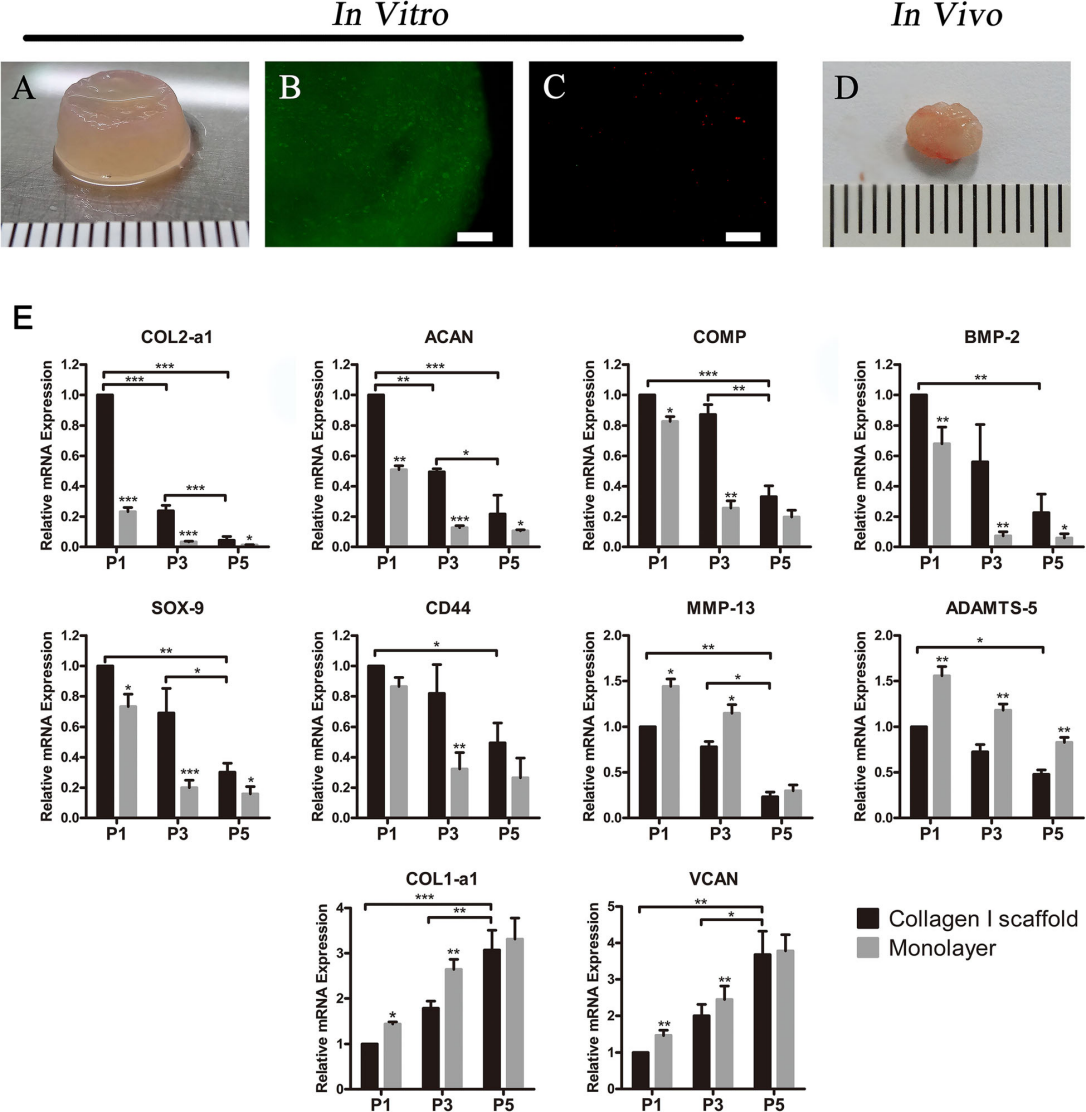
Characteristics of type I collagen scaffolds and phenotypic analysis of OA chondrocytes during 3D culture in collagen I scaffolds. **a** General view of collagen I scaffolds in vitro. **b** Live cell staining of chondrocytes in type I collagen scaffolds. Scale bar = 1 mm. **c** Dead cell staining of chondrocytes in type I collagen scaffolds. Scale bar = 1 mm. **d** Gross morphology of type I collagen scaffold constructs after implantation. **e** P1, P3, and P5 OA chondrocytes were seeded into type I collagen scaffolds for 3D culture at a seeding density of 2 × 10^6^ cells/ml. After 2 weeks of in vitro culture, gene expression profiles of different sets of OA chondrocytes in type I collagen scaffolds and monolayer culture were assessed (*n* = 3 in each group). *p < 0.05, **p < 0.01, and ***p < 0.001; data are mean ± standard

**Fig. 3:**
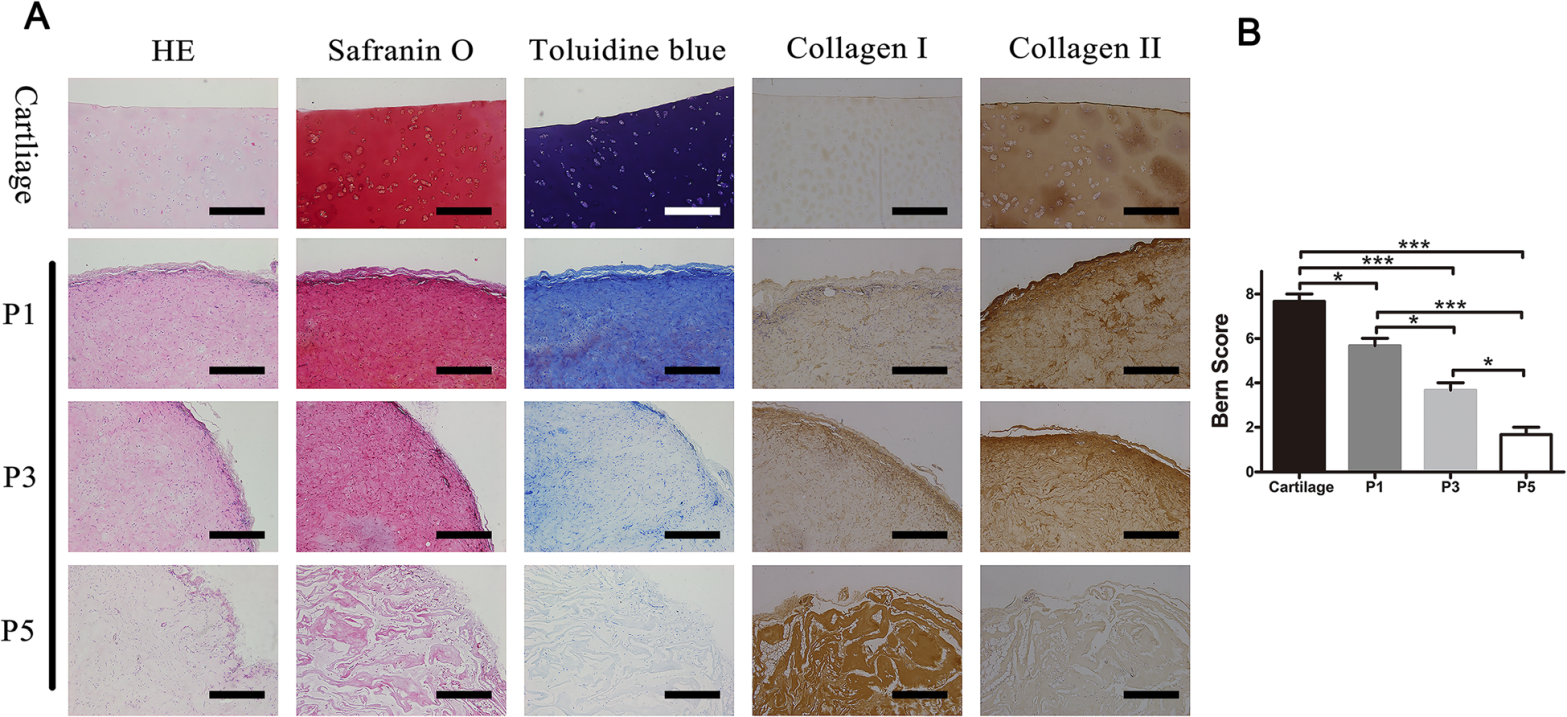
The effects of cellular phenotype on the chondrogenic capacity in vivo. **a** In vivo chondrogenic capacity of OA chondrocytes inoculated in type I collagen scaffolds. P1, P3, and P3 chondrocytes (2 × 10^6^ cells/mL) were seeded into type I collagen scaffolds were implanted subcutaneously in SCID mice for 4 weeks to establish a model of in vivo ectopic cartilage formation. The samples were analyzed by hematoxylin and eosin (H&E), toluidine blue (TB) staining, safranin O(SO) staining, and immunofluorescence staining for type II and I collagen. Scale bar = 1 mm. **b** Bern score evaluation (*n* = 3 in each group). *p < 0.05, **p < 0.01, and ***p < 0.001; data are mean ± standard

**Fig. 4:**
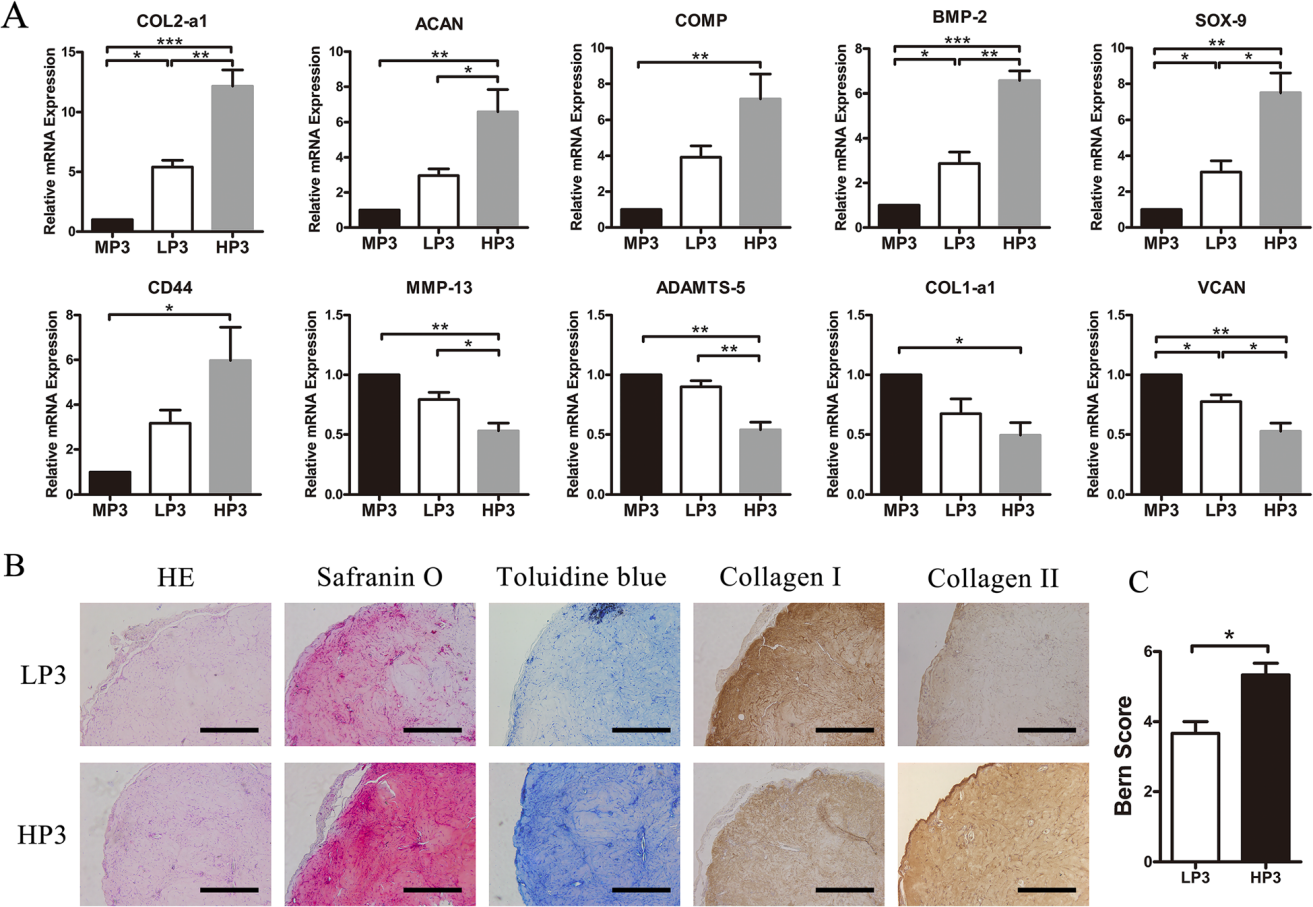
Effects of cellular density on the expression of specific genes and in vivo chondrogenic capacity of OA chondrocytes in type I collagen scaffolds. **a** Gene expression profiles of low (5 × 10^5^ cells/ml) and high (2 × 10^6^ cells/ml) density OA chondrocytes in type I collagen scaffolds (*n* = 3 in each group). **b** Histological analysis of scaffolds with different OA chondrocyte seeding densities. Scale bar = 1 mm. **c** Bern score evaluation (*n* = 3 in each group). *p < 0.05, **p < 0.01, and ***p < 0.001; data are mean ± standard. MP3, monolayer cultured P3; LP3, low-density P3 in type I collagen scaffolds; HP3, high-density P3 chondrocytes in type I collagen scaffolds

**Fig. 5:**
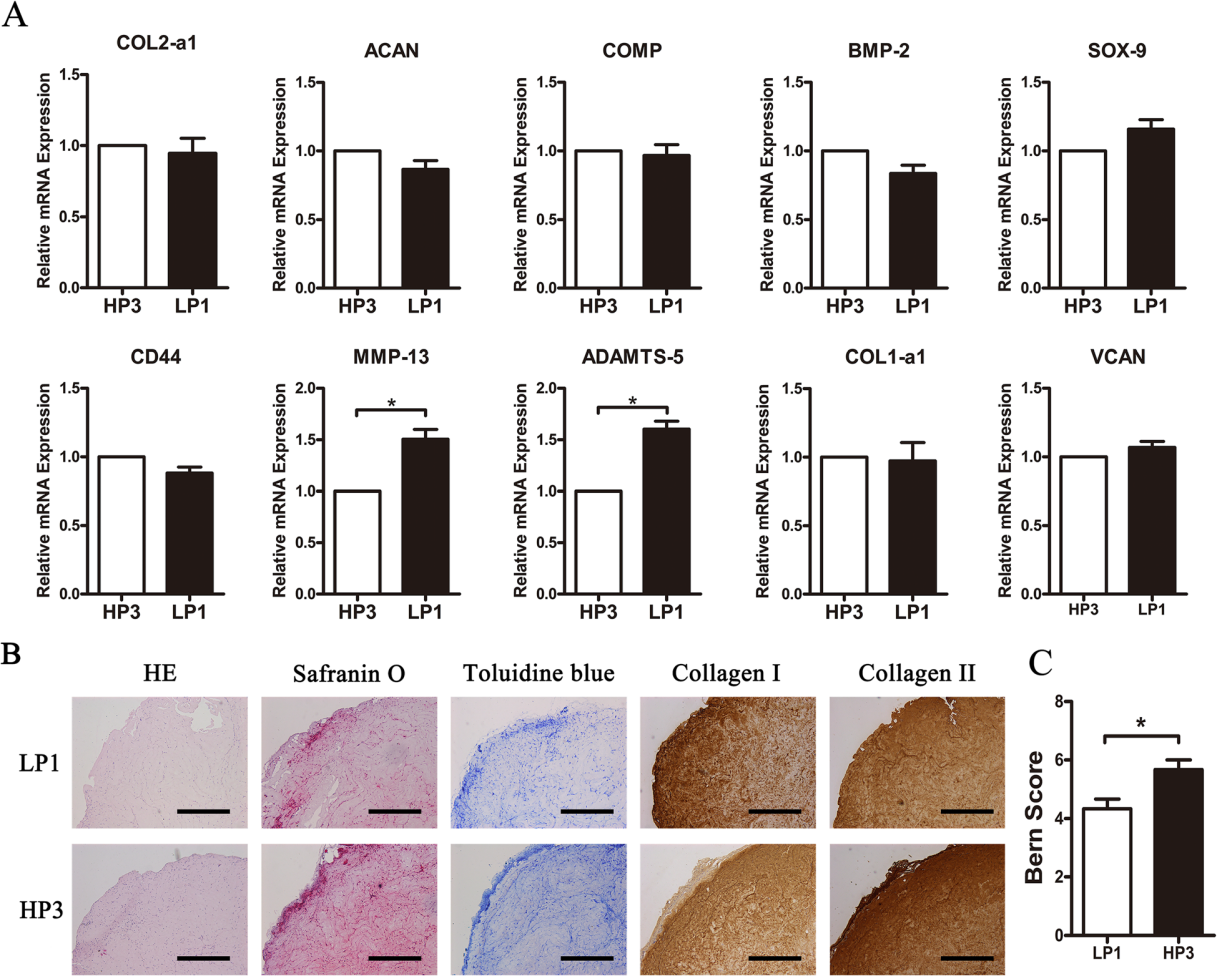
Expression of specific genes and in vivo chondrogenic capacity of LP1 and HP3. **a** Gene expression profiles of LP1 (5 × 10^5^ cells/ml) and HP3 (2 × 10^6^ cells/ml) OA chondrocytes in type I collagen scaffolds (*n* = 3 in each group). **b** Histological analysis of scaffolds. Scale bar = 1 mm. **c** Bern score evaluation (*n* = 3 in each group). *p < 0.05, **p < 0.01, and ***p < 0.001; data are mean ± standard. LP1, low-density P1 in type I collagen scaffolds; HP3, high-density P3 chondrocytes in type I collagen scaffolds

Statistical analysis was performed with SPSS 20.0 (IBM Corp, Chicago, USA). Data were presented as mean ± standard deviation. Statistical comparisons of two independent groups (Fig. 4c, Fig. 5a, and Fig. 5c) were made using the Shapiro–Wilk test for normality, Levene’s test for homogeneity of variance, and the two-tailed independent *t* test. Multiple comparisons (Fig. 1a, c, Fig. 3b, and Fig. 4a) were performed using the Shapiro–Wilk test, Levene’s test, and one-way analysis of variance (ANOVA) with post hoc Bonferroni test. Multiple comparisons (Fig. 2e) were performed using the Shapiro–Wilk test, Levene’s test, and two-way analysis of variance (ANOVA) with post hoc Bonferroni test. Each *n* indicates the number of biologically independent samples. *P* < 0.05 was considered statistically significant.

## Results

### Expansion of OA chondrocytes in monolayer culture

Differences in gene expression patterns among monolayer cultured P1, P3, and P5 OA chondrocytes were examined (Fig. 1a). With the passage of cells, mRNA expression levels of COL2a1, ACAN, COMP, and SOX9 were dramatically decreased between P1, P3, and P5. mRNA expression levels of BMP2 and CD44 were decreased between P1 and P5. Interestingly, the expression levels of matrix proteinases MMP13 and ADAMTS5 were also decreased during monolayer culture, while COL1a1 and VCAN amounts were significantly increased from P1 to P5.

Extracellular matrix (ECM) production was examined by histochemical and immunofluorescent staining (Fig. 1b). In OA chondrocytes cultured in monolayer, with the passage of cells, proteoglycan amounts (determined by SO and TB staining) were markedly reduced. P1 showed the strongest staining signals for collagen II among the three passages, and this protein was rarely observed in P5. In contrast, type I collagen was rarely found in P1 and P3, and the strongest signals were recorded in P5. These findings indicated that P5 cells lost the chondrocyte phenotype. In addition, the accumulation of GAG per 5 × 10^5^ cells also gradually decreased from P1 to P5 (Fig. 1c).

## Effects of cellular phenotype on the re-differentiation potential and chondrogenic capability of OA chondrocytes cultured in the type I collagen scaffold

### Re-differentiation potential of OA chondrocytes

To evaluate the re-differentiation potential of expanded OA chondrocytes, cells with different phenotypes (P1, P3, and P5) were seeded into the type I collagen scaffold at high seeding density (2 × 10^6^ cells/ml) for 2 weeks of 3D culture in vitro that mimics the ACI environment (Fig. 2a). The cell viability assay showed that the expanded OA chondrocytes(only showed P3)grew well and had good activity in the type I collagen scaffold (Fig. 2b, c); this proved that the scaffold material is not cytotoxic.

First, observed the difference between the monolayer culture group and the 3D culture group (Fig. 2e), for P1 and P3 cells; the gene expression levels of specific chondrogenic factors, such as COL2a1, ACAN, COMP, SOX-9, BMP-2, and CD44 in 3D-cultured groups were all reversed and significantly higher than the values obtained for monolayer cultures, and the expression levels of COL1a1, VCAN, MMP13, and ADAMTS5 were significantly lower. However, for P5 OA chondrocytes, only COL2a1, ACAN, BMP2, and SOX9 mRNA levels were reversed with a statistically significant difference between the monolayer and 3D environment; no significant differences in COMP, CD44, COL1a1, VCAN, MMP13, and ADAMTS5 amounts were detected. This showed that P1 and P3 chondrocytes can effectively re-differentiate during the 3D culture of type I collagen scaffolds, and had better re-differentiation capabilities, while P5 chondrocytes had very limited re-differentiation capabilities.

Next, only observed the differences between the 3D culture groups of P1, P3, and P5 cells (Fig. 2e). Interestingly, when cultured on the collagen I scaffold, the expression levels of COMP, BMP2, SOX9, CD44, MMP13, ADAMTS5, COL1a1, and VCAN showed significant differences between 3D-P1 and 3D-P5 cells, while no significant differences were obtained between 3D-P1 and 3D-P3. But these differences did exist between monolayer-cultured P1 and P3 (Fig. 1a), and this showed that under 3D culture conditions, although the difference in gene expression caused by chondrocyte de-differentiation still exists, it effectively delays the process of dedifferentiation of articular chondrocytes.

### Chondrogenic capability of OA chondrocytes

To assess the chondrogenic capability of expanded OA chondrocytes, after 24 h cultured in vitro, we implanted the type I collagen scaffold with different phenotypes (P1, P3, and P5) into SCID mice for 4 weeks (Fig. 2d). In vivo data revealed that P1-collagen I scaffold constructs were similar to the normal cartilage, while the tissue formed by the P5-collagen I scaffold became fibrotic. Moreover, staining intensities of safranin-O, toluidine blue, and anti-collagen II in these tissues also decreased from P1 to P5. In contrast, the staining intensity of anti-collagen I was very high in the P5-collagen I scaffold, while the P1-collagen I scaffold and the normal cartilage were essentially negative (Fig. 3a). These findings were confirmed by histological scoring according to the Bern grading system (Fig. 3b). The natural cartilage had the highest score, and the score gradually decreased with de-differentiation of the cell phenotype (P1 to P5).

## Effects of cell seeding density on re-differentiation potential and chondrogenic capability of OA chondrocytes cultured in the type I collagen scaffold

### Low versus high seeding density of P3 cells

After 2 weeks of culture in vitro, the differences in gene expression patterns were examined among low seeding density of P3 (LP3, 5 × 10^5^ cells/mL), high seeding density of P3 (HP3, 2 × 10^6^ cells/mL) in collagen I scaffolds, and monolayer P3 cells (MP3) (Fig. 4a). Cells cultured in monolayer expressed only basal levels of COL2a1, ACAN, COMP, BMP2, CD44, and SOX9. Conversely, those cultured in type I collagen scaffolds began to express or showed significantly increased amounts of chondrogenic genes, especially at high cell seeding density. HP3 expressed higher levels of COL2a1, ACAN, SOX9, and BMP2 compared with LP3, and the expression levels of VCAN, MMP13, and ADAMTS5 were significantly lower than those of LP3. Although LP3 expressed more COL2a1, SOX9, BMP2, and VCAN than MP3, there were no significant differences in the expression levels of ACAN, CD44, COMP, MMP13, ADAMTS5, and COL1a1.

In vivo chondrogenic capability at different seeding densities of OA chondrocytes (LP3-collagen I and HP3-collagen I scaffolds) were examined by histological and immunohistochemical staining (Fig. 4b) after implanted into SCID mice for 4 weeks. Safranin-O, toluidine blue, and anti-collagen II staining signals showed that HP3-collagen I scaffolds were stronger than the LP3 group, in agreement with Bern grading system scores (Fig. 4c).

### Low seeding density of P1 versus high seeding density of P3

High seeding density of P3 (HP3, 2 × 10^6^ cells/mL) and low seeding density of P1 (LP1, 5 × 10^5^ cells/mL) were implemented in type I collagen scaffolds for 2 weeks in in vitro 3D cultures. Cartilage-specific gene expression profiles were basically identical between the two groups, except for MMP13 (*p* = 0.032) and ADAMTS5 (*p* = 0.036) (Fig. 5a).

As for chondrogenic capability, in vivo histological and immunohistochemical staining data showed that HP3-collagen I scaffolds could maintain a better cellular phenotype, with slightly stronger staining intensity of safranin-O, toluidine blue, and anti-collagen II compared with LP1-collagen I scaffolds (Fig. 5b). The results were in agreement with Bern grading system scores (Fig. 5c).

## Discussion

Chondrocytes are widely used in cartilage tissue engineering, but there are still some challenges to be overcome [11]. Currently, the major challenges in chondrocyte-based cartilage engineering include cell re-differentiation capacity and expansion procedures, and the range of chondrocyte quantities against lesion volume in triggering hyaline cartilage tissue formation [12]. The use of OA patients’ own chondrocytes for MACI in treating cartilage damage caused by OA is required to solve these problems.

In the current study, we demonstrated that mRNA levels of fibrotic genes (COL1a1 and VCAN) were gradually increased, while the amounts of chondrogenic genes (COL2a1, ACAN, SOX9, BMP2, CD44, and COMP) were progressively decreased in OA chondrocytes from P1 to P5 during monolayer culture. The differences in gene expression patterns were confirmed at the protein level, with higher signals of collagen type II and GAG in P1 as well as elevated collagen type I in P5, consistent with the well-known de-differentiation phenomenon [13]. Meanwhile, the expression levels of matrix proteinases (MMP-13 and ADAMTS-5) were decreased, indicating that OA chondrocytes had less degradation activity during monolayer culture.

To be able to use MACI for treating cartilage defects in OA patients, it is highly important to investigate whether monolayer expanded OA chondrocytes recover the chondrogenic phenotype when cultured in the 3D environment. Using collagen as a scaffold material, cells interact either directly with the fibers via integrin receptors or via the autogenous matrix, which triggers cellular development and matrix deposition [14]. Porous collagen-based scaffolds have been widely used for chondrocyte transplantation in vivo [15]. Kanda Chaipinyo et al. biopsied cartilage from two young autopsies (aged 21 and 25) and seeded at low density in type I collagen gels which demonstrates that chondrocytes cultured at low density in type I collagen gels can be used in the ACI procedure as they provide a sufficient number of cells which synthesize a cartilage-like matrix [16]. But they did not prove that OA chondrocytes can have the same effect. The current study demonstrated the ability of type I collagen scaffolds to support the re-differentiation of OA chondrocytes after de-differentiation and promote phenotypic stability, reflected by increased COL2a1, ACAN, COMP, SOX9, and BMP2 expression levels and decreased COL1a1, VCAN, MMP13, and ADAMTS5 amounts. This indicates that autologous chondrocytes from OA patients constitute a potential cell source for treatment.

While MACI has shown improvements over past therapeutic tools, biomechanics and long-term integration remain problematic, possibly due to the de-differentiation of chondrocytes prior to scaffold-seeding. The notion of chondrocyte quality control was introduced, suggesting that the enriched stable population of chondrocytes could be used for more reproducible results of ACI in 2001 [17]. Although the 3D culture environment can induce de-differentiated chondrocytes to undergo re-differentiation, it is not suitable for any phenotype chondrocytes. In this study, chondrocytes with improved cell phenotypes (P1 and P3) clearly retained the chondrogenic phenotype and produced more collagen type II and GAG, while poor-quality chondrocytes (P5) could not. Schulze-Tanzil et al. showed that 1–4 generation chondrocytes induce re-differentiation after 3D culture on alginate beads, but limited re-differentiation of chondrocytes was found for 5–8 generations [18]. The differentiation state of chondrocytes, which reflects their quality, has significant effects on the outcome of ACI [19]. The present study supports this idea by showing that P5 chondrocytes produced a mechanically inferior neo-tissue when seeded in collagen I scaffolds, which probably would not be sufficient for clinical application. The above results indicated that phenotypically superior chondrocytes seeded in collagen scaffolds could maintain an enhanced phenotype and produce more robust cartilage-like ECM compared with phenotypically inferior cells.

Similarly, cell density was also a key factor in chondrocyte differentiation, because proper cell-cell contact is necessary for differentiation and optimal matrix deposition. Studies assessing tissue biopsies in postoperative years showed that articular cartilage tissue repair is very limited, reflected by a scarcity of chondrocytes and the ECM not reaching the hardness of undamaged cartilage [10]. This may be due to differences in cell number and implantation density. Silvia et al. found that high-seeding density of cells in 3D scaffolds is more critical for the generation of cartilaginous constructs than the stage of cell differentiation reached following expansion [20]. In the current study, we inoculated P3 chondrocytes into collagen I scaffolds at 5 × 10^5^ cells/ml and 2 × 10^6^ cells/ml and comparatively assessed the latter with monolayer expansion. We found that HP3 showed more stable chondrogenic phenotypes and produced more collagen type II and GAG compared with LP3. This indicated that cell density in the 3D culture system plays a significant role in maintaining chondrocyte differentiation. High-density chondrocytes combined with type I collagen scaffolds can effectively slow the de-differentiation process and maintain the chondrocyte phenotype. However, the planting density of cells has a ceiling effect. Olderøy et [21]. analyzed the chondrogenic differentiation of hMSCs in alginate at different seeding densities, which were all above those used in this study (10 × 10^6^, 25 × 10^6^, and 50 × 10^6^ cells/cm^3^). At such high cell densities, gene expression of the chondrogenic markers Col2 and ACAN did not further increase.

However, in order to obtain large amounts of chondrocytes, multiple passage expansion cultures are required, which leads to de-differentiation and influences the phenotype of chondrocytes [22]. The quality and density are therefore conflicting variables in chondrocyte expansion, and it is worth discussing which one is more important. We found that the expression profiles were basically the same between the HP3 and LP1 groups in vitro. While in vivo, HP3-collagen I scaffolds had better chondrogenic capacity than the LP1 group and could form better cartilage-like tissues with stronger staining signals for safranin-O, toluidine blue, and anti-collagen II which were in agreement with Bern grading system scores. These results indicated that for chondrocytes with worse phenotype(P3 OA chondrocytes), increasing cell density could effectively compensate for gene expression differences observed with chondrocytes with a better phenotype (P1 OA chondrocytes). This effect of density on cell differentiation, known as quorum sensing, is well documented in prokaryotes and was recently reported in vertebrates [23]. A balance must be met between increasing proliferation and preventing de-differentiation. The goal of cartilage repair requires the chondrocytes not only to reach an appropriate density but also to generate an ECM with the appropriate texture. Although the experimental results show that the use of P1 chondrocytes is better than P3 chondrocytes, in actual clinical applications, the number of chondrocytes removed from patients is limited. When expanded to P1 in vitro, sufficient cell numbers cannot be achieved. However, when it is expanded to P3, it can reach a considerable number of cells to meet high-density chondrocyte transplantation, and our experimental results show that P3 chondrocytes can meet clinical needs. In short, P3 chondrocytes reach a balance between cell mass and cell number, which can meet the needs of practical clinical applications.

Based on the above discussion, we can know that the use of cartilage transplantation in young patients to repair local cartilage damage has achieved good results, but when patients have OA symptoms, whether this method is effective or not has not been studied in depth. Our research has gathered to explore this point. Although the chondrocytes of OA patients are not normal chondrocytes, when they were implanted at high density in type I collagen scaffolds, they showed good behavior of cartilage-forming ability both in vitro and in vivo and secreted more normal cartilage extracellular matrix.

## Conclusions

In conclusion, freshly isolated chondrocytes from OA patients could be a source of replacement for articular chondrocytes being commonly used in MACI and have positive implications for patients suffering from an early onset osteoarthritis. The cellular phenotype and seeding density of OA chondrocytes loaded on collagen I scaffolds are important factors affecting the outcome. Implantation of phenotypically superior OA chondrocytes in collagen I scaffolds at high density could be a promising tool for the treatment of elderly OA patients.

## Data Availability

The datasets used and/or analyzed during the current study are available from the corresponding author on reasonable request.

## Abbreviations

DMMB: Modified 1,9-dimethyl-methylene blue
ECM: Extracellular matrix
MACI: Matrix-associated autologous chondrocyte implantation
OA: Osteoarthritis
